# Clinicopathological roles of adiponectin and leptin receptors in endometrial carcinoma

**DOI:** 10.3892/ol.2014.1846

**Published:** 2014-01-31

**Authors:** HIROMITSU YABUSHITA, KEITA IWASAKI, YUKIHIKO OBAYASHI, AKIHIKO WAKATSUKI

**Affiliations:** Department of Obstetrics and Gynecology, Aichi Medical University, School of Medicine, Nagakute, Aichi 480-1195, Japan

**Keywords:** endometrial carcinoma, adiponectin receptor, leptin receptor, immunohistochemistry

## Abstract

To clarify the roles of adiponectin receptor (AdipoR) and leptin receptor (ObR) in endometrial carcinoma, the expression of AdipoR-1 and -2 and ObR in endometrial cancer was examined immunohistochemically, and correlations with clinicopathological implications were also analysed. Paraffin-embedded tissues were obtained from 77 patients with endometrial carcinoma and were stained immunohistochemically using antibodies against AdipoR-1, AdipoR-2 and ObR. AdipoR-1, AdipoR-2 and ObR were localised predominantly in the cell membrane and cytoplasm of tumour cells and normal endometrial cells. In 77 cases of endometrial cancer, positive expression was observed in 46 cases (59.7%) for AdipoR-1, 47 cases (61.0%) for AdipoR-2 and 33 cases (42.9%) for ObR. Expression of AdipoR-1 was observed most in stage I cases, G1 tumours, tumours with shallow myometrial invasion, tumours negative for lymphovascular space involvement, cases negative for adnexal invasion and cases with no lymph node metastasis. However, the expression of AdipoR-2 and ObR showed no correlation with any clinicopathological factors. Kaplan-Meier analyses revealed that progression-free and overall survival times were longer in cases with positive AdipoR-1 expression compared with negative AdipoR-1 expression. Poor expression of AdipoR-1, thus, appears to be associated with tumour grade, myometrial invasion, adnexal invasion, lymph-vascular space involvement and lymph node metastasis, as well as poor prognosis, in endometrial cancer.

## Introduction

Endometrial cancer is showing rapid increases in mortality and incidence in Japan (1). Previous clinicopathological studies have shown that poor prognosis of endometrial cancer is associated with the histological grade of the tumour, presence of malignant cells in ascites or the washing of specimens for cytology, cervical invasion of malignant cells, deep myometrial invasion of malignant cells, lymph node metastasis and involvement of malignant cells in the lymphovascular space (2–4).

Obesity is a well-established risk factor associated with several types of cancer, including endometrial cancer (5–8). The factor linking obesity to endometrial cancer appears to be excessive exposure to various factors produced by adipose tissue. Adipose tissue is a source of oestrogen, insulin and insulin-like growth factors, all of which are considered to be involved in endometrial tumourigenesis (9,10). In addition to these factors, adipose tissues produce various bioactive hormones called adipokines, the most prominent of which are leptin and adiponectin. These may significantly influence the growth and proliferation of tumours (11–13).

Leptin, a product of the obese (Ob) gene, is produced predominantly in adipose tissue and expressed in normal tissues and malignant breast tissues (14–16). Expression of leptin and leptin receptor (ObR) is more frequent in carcinoma than in normal tissue (17,18). Leptin is involved in a variety of functions, including appetite regulation, bone formation, reproduction and angiogenesis (19), and may affect processes associated with cancer initiation and progression, resulting in the development of metastases (15,17,20,21). Leptin acts through ObR, which is encoded by the Ob gene. Binding of leptin to ObR activates the Janus kinase (JAK)/signal transducer and activator of transcription signalling pathway and induction of JAK stimulates phosphoinositol-3-kinase (PI3-kinase). Activation of PI3-kinase increases cell migration and invasion via the Rac/Rho pathways and stimulates the major growth and survival Akt pathway (13).

Adiponectin is an additional type of adipocytokine, predominantly secreted by adipocytes (12). In contrast to other adipocytokines, adiponectin levels correlate inversely with central fat accumulation (22). Adiponectin may exert antineoplastic activity through suppression of tumour proliferation and neoangiogenesis, and through induction of apoptosis (23–25). In addition, an inverse correlation has been found between plasma adiponectin levels and the histological grade of endometrial cancer (26). However, the precise effects of adiponectin in endometrial cancer remain unclear. In total, two adiponectin receptors (AdipoR-1 and AdipoR-2) have been identified and *in vitro* studies have shown that the two AdipoR genes are expressed in human monocytes, macrophages, adipocytes, vascular smooth muscles, a primary osteoblast cell line, pancreatic cells and other cell types (27–30). Previous *in vivo* studies have shown that AdipoR-1 is abundantly expressed in skeletal muscle and the liver, whereas AdipoR-2 is predominantly expressed in the liver. Additionally, AdipoR-1 in the liver activates AMP-activated protein kinase pathways, while AdipoR-2 activates peroxisome proliferator-activated receptor pathways (27,31,32). In addition, expression of AdipoR has previously been documented in several human cancer cell lines (33,34).

The aim of the present study was to determine whether the immunohistochemical expression of AdipoR-1, AdipoR-2 or ObR correlates with the clinicopathological manifestations and clinical outcomes of endometrial carcinoma patients.

## Materials and methods

### Clinical samples

Formalin-fixed, paraffin-embedded tumour tissues were obtained from 77 patients with endometrioid adenocarcinoma of the endometrium. All patients attended the Gynaecology Clinic at the Aichi Medical University Hospital (Nagakute, Japan) and were diagnosed with endometrial endometrioid adenocarcinoma based on post-surgical assessment. Clinicopathological characteristics of the patients are shown in Table I. Normal endometrial tissue was also obtained from 19 patients with benign uterine disease (uterine leiomyoma, n=12; and uterine prolapse, n=7). All study protocols were approved by the regional ethics committee of the Aichi Medical University, School of Medicine (Nagakute, Japan). Written informed consent was obtained from all participants prior to study enrolment.

### Immunohistochemistry

The prepared 3-μm sections were deparaffinised and rehydrated. Following microwave processing for 25 min in 10 mM citrate buffer (pH 6.0), sections were incubated for 30 min in methanol containing 0.5% H_2_O_2_. Following incubation in normal goat serum for 1 h at room temperature to block non-specific staining, slides were incubated overnight with the primary antibodies at 4°C. The primary antibodies used were rabbit anti-human AdipoR-1 (raised against amino acid residues 357–375) antiserum, rabbit anti-human AdipoR-2 (raised against amino acid residues 374–386) antiserum (both purchased from Phoenix Pharmaceuticals, Burlingame, CA, USA) and rabbit anti-human ObR (raised against amino acid residues 541–840) antiserum (Santa Cruz Biotechnology, Inc., Santa Cruz, CA, USA), used at dilutions of 1:500, 1:200 and 1:200, respectively. Anti-rabbit antibody-labelled polymer horseradish peroxidase (ChemMate Envision kit; Dako Japan, Kyoto, Japan) was used as the secondary antibody, applied for 30 min at room temperature. The horseradish peroxidase reaction was developed with 3,3′-diaminobenzidine tetrahydrochloride (Katayama Chemical Industries Co. Ltd., Osaka, Japan)and sections were counterstained with haematoxylin (Katayama Chemical Industries Co. Ltd.) for microscopic examination (Olympus BX50, Olympus Corp., Tokyo, Japan). Sections were defined as showing positive expression when >50% of tumour cells were intensely stained.

### Statistical analysis

The statistical significance of differences among different categories of expression was analysed using the χ^2^ test and two-way analysis of variance. Progression-free and overall survival were analysed using the Kaplan-Meier method and log-rank test, and the potential significance of multiple prognostic factors for progression-free and overall survival were analysed by the Cox proportional hazard regression test. P<0.05 was considered to indicate a statistically significant difference.

## Results

### AdipoR-1, AdipoR-2 and ObR localisation

**Figure 1:**
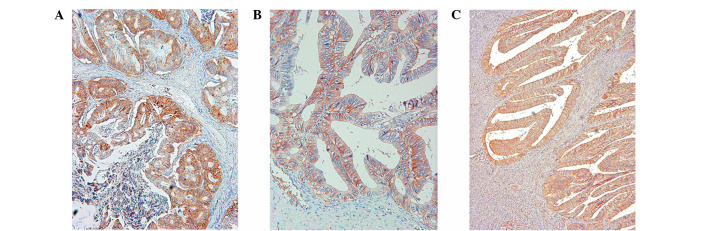
Representative examples of immunohistochemical visualisation of AdipoR-1, AdipoR-2 and ObR in endometrial carcinoma. (A) AdipoR-1 expression in G2 endometrioid adenocarcinoma and (B) AdipoR-2 and (C) ObR expression in G1 endometrioid adenocarcinoma. AdipoR-1, adiponectin receptor type 1; AdipoR-2, adiponectin receptor type 2; ObR, leptin receptor. Magnification, ×200.

AdipoR-1, AdipoR-2 and ObR were localised predominantly to the cell membrane and cytoplasm of tumour cells (Fig. 1). In normal endometrial tissue, the expression of AdipoR-1, AdipoR-2 and ObR was identified in endometrial glandular cells, in the proliferative and secretory phases. Expression of AdipoR-1 was found in nine of the 10 proliferative-phase specimens and in all nine secretory-phase specimens. In addition, the expression of AdipoR-2 and ObR were found in all 10 proliferative-phase and nine secretory-phase specimens.

### AdipoR-1, AdipoR-2 and ObR expression

In the 77 endometrial cancer cases, positive expression was observed in 46 cases (59.7%) for AdipoR-1, 47 cases (61.0%) for AdipoR-2 and 33 cases (42.9%) for ObR. Expression of AdipoR-1 was observed most in stage I cases versus stage II or III cases, G1 tumours versus G2 or G3 tumours, tumours with shallow myometrial invasion versus tumours with deep myometrial invasion, tumours with negative lymphovascular space involvement versus tumours with positive lymphovascular space involvement, cases with negative adnexal invasion versus cases with positive adnexal invasion and cases with negative lymph node metastasis versus cases with positive lymph node metastasis. Conversely, cervical stromal invasion, peritoneal cytology and body mass index exhibited no correlation with AdipoR-1 expression (Table II). However, expression of AdipoR-2 appeared unrelated to any clinicopathological factors and expression of ObR was unrelated to any factors other than tumour grade.

### Survival rates

**Figure 2:**
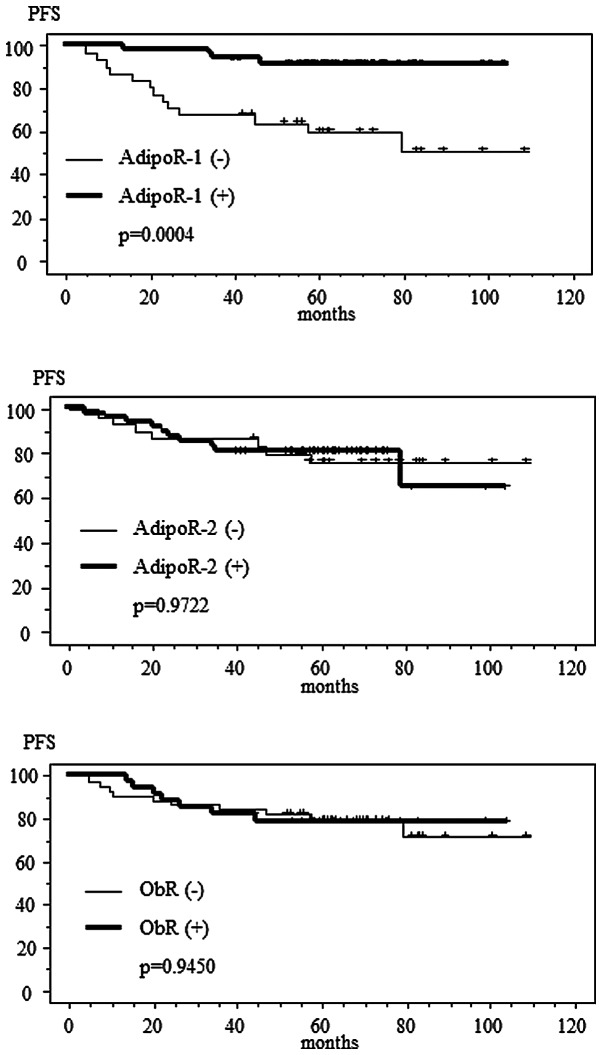
PFS was analysed using the Kaplan-Meier method and compared in relation to AdipoR-1, AdipoR-2 and ObR expression. PFS, progression-free survival; AdipoR-1, adiponectin receptor type 1; AdipoR-2, adiponectin receptor type 2; ObR, leptin receptor.

**Figure 3:**
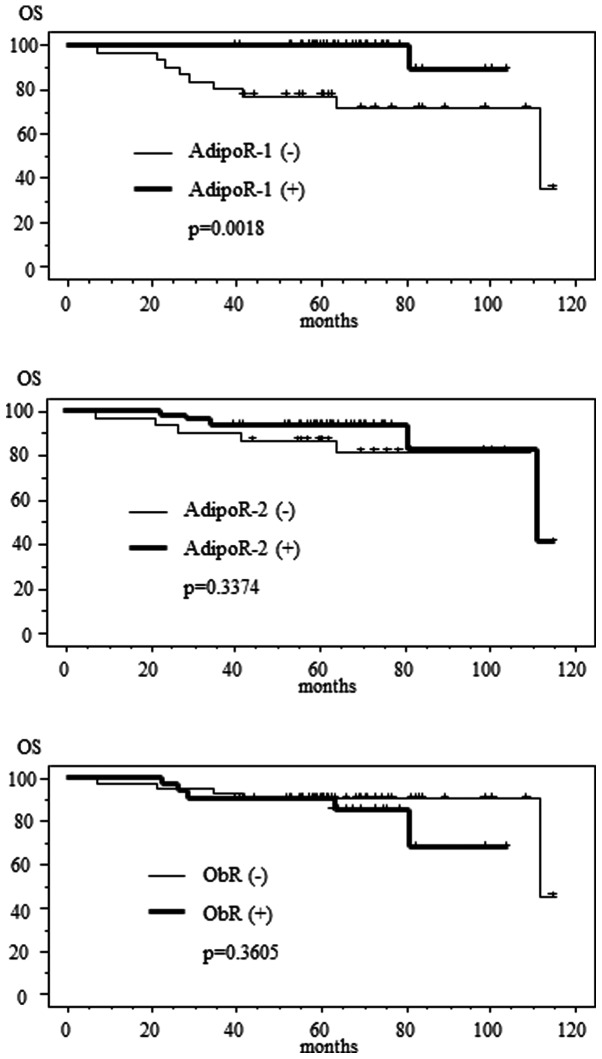
OS was analysed using the Kaplan-Meier method and compared in relation to AdipoR-1, AdipoR-2 and ObR expression. OS, overall survival; AdipoR-1, adiponectin receptor type 1; AdipoR-2, adiponectin receptor type 2; ObR, leptin receptor.

In the Kaplan-Meier analyses, progression-free and overall survival times were longer in cases with positive AdipoR-1 expression compared with negative AdipoR-1 expression, while survival time did not show any correlation with AdipoR-2 or ObR expression (Figs. 2 and 3; Tables III and IV).

### Univariate and multivariate analyses

Univariate logistic regression analysis revealed that advanced-stage disease, high tumour grade, deeper myometrial invasion, cervical stromal invasion, adnexal invasion, involvement of the lymphovascular space, lymph node metastasis and negative AdipoR-1 expression were all significantly associated with poor progression-free survival. Similarly, multivariate Cox proportional hazard regression analysis revealed adnexal invasion and lymph node metastasis as independent variables associated with shorter progression-free survival (Table III). Univariate logistic regression analysis revealed that advanced-stage disease, high tumour grade, deeper myometrial invasion, cervical stromal invasion, adnexal invasion, lymph node metastasis and negative AdipoR-1 expression were significantly associated with poor overall survival, while multivariate Cox proportional hazard regression analysis revealed that adnexal invasion and lymph node metastasis were also identified as independent variables associated with shorter overall survival (Table IV).

## Discussion

Obesity is a known risk factor for endometrial cancer (8). Adipose tissues produce various bioactive substances called adipokines, the most prominent of which are leptin and adiponectin. Leptin has been found to positively correlate with obesity and is a well-known regulator of food intake and energy balance. By reducing tissue sensitivity to insulin, leptin is responsible for hyperinsulinaemia (35). Adiponectin has been found to negatively correlate with obesity, and low levels of adiponectin have been shown to have a high correlation with hyperinsulinaemia and the degree of insulin resistance independent of adiposity (36). These adipokines are reportedly associated with carcinogenesis and tumour growth in several types of cancer, including breast, colon, stomach and endometrial cancer (5–8). Serum concentrations of leptin are reportedly higher, and those of adiponectin lower, in patients with endometrial cancer compared with the control subjects (26,37–40). The balance of leptin and adiponectin levels in individuals, rather than leptin or adiponectin levels alone, may indicate such physiological changes as the development of endometrial cancer (41).

Previously, adiponectin has been shown to exert suppressive effects on tumour development (42). Low adiponectin serum concentrations have been identified in patients affected by cancer (38,42–44), including endometrial cancer. Serum adiponectin levels have been found to be inversely and independently associated with endometrial cancer (39).

The actions of adiponectin are mediated by binding to two receptors, AdipoR-1 and AdipoR-2 (31), and leptin actions are also mediated by binding to ObR.

Adiponectin has been previously reported to suppress endometrial cancer proliferation through AdipoRs and also increase the expression of the adaptor molecule, *LKB1*, which is required for adiponectin-mediated activation of the AMPK/S6 axis and modulation of cell proliferation, colony formation, adhesion and invasion of endometrial carcinoma cell lines *in vitro* (34,45). In addition, adiponectin reportedly acts to potently inhibit endothelial cell proliferation and migration *in vitro* and, in chick chorioallantoic membrane and mouse corneal angiogenesis assays, adiponectin shows marked activity in preventing new blood vessel growth. Furthermore, the antiendothelial mechanisms have been demonstrated to involve the activation of caspase-mediated endothelial cell apoptosis. Adiponectin induces the cascade activation of caspase-8, -9 and -3, leading to cell death. In a previous mouse tumour model, adiponectin significantly inhibited primary tumour growth (23). Impaired tumour growth appeared to be associated with decreased neovascularisation, leading to significantly increased tumour cell apoptosis. These results demonstrated the induction of endothelial apoptosis as an unique mechanism for adiponectin-induced antiangiogenesis. Adiponectin, as a direct endogenous angiogenesis inhibitor, may have therapeutic implications in the treatment of angiogenesis-dependent diseases, including endometrial cancer (23).

The expression of these receptors has previously been documented in normal endometrium (46) and endometrial cancer tissues (47), but the role of AdipoR-1, AdipoR-2 and ObR in endometrial carcinoma has not been fully determined.

The present study demonstrated that AdipoR-1, AdipoR-2 and ObR are expressed in endometrial carcinoma tissue, and that AdipoR-1 expression inversely correlates with high histological grade, deep myometrial invasion, involvement of the lymphovascular space, adnexal invasion and lymph node metastasis, and is associated with improved progression-free and overall survival. However, AdipoR-2 and ObR expression was not found to correlate with any clinicopathological factors or survival in patients with endometrial carcinoma. Immunohistochemical expression of adiponectin in paired samples of endometrial carcinoma was also examined, but failed to observe any adiponectin expression in tumour cells or stromal elements (data not shown). Thus, the present results suggested that autocrine and paracrine stimulations of AdipoRs are unsuitable for endometrial carcinoma.

Previously, Yamauchi *et al* (47) reported that decreased AdipoR-1 expression significantly correlates with higher histological grade, myometrial invasion and lymph node metastasis of endometrioid adenocarcinoma. These results raised the possibility that the decreased expression of AdipoRs is implicated in the development, invasion and metastasis of human endometrioid adenocarcinoma. Thus, AdipoRs may be considered as therapeutic targets for endometrioid adenocarcinoma. In AdipoR-positive endometrioid adenocarcinoma, adiponectin-based anticancer therapy may prove useful. In addition, Yamauchi *et al* (47) commented that AdipoR-1 and AdipoR-2 may be considered as therapeutic targets for endometrioid adenocarcinomas. Methods leading to the upregulation of AdipoRs or development of specific AdipoR agonists (such as osmotin) may prove beneficial in the treatment of endometrioid adenocarcinomas, and the results of the current study appear to support this perspective.

Several limitations must be considered when interpreting the results of the present study. Firstly, the immunohistochemical expression of AdipoR-1, AdipoR-2 and ObR were analysed, but gene and protein expression levels were not revealed in endometrial carcinoma. Secondly, the present study was unable to assume that correlations exist between serum adiponectin or leptin levels and the expression of AdipoRs and ObR in patients with endometrial carcinoma. Despite these limitations, we considered the present observations to be meaningful, since they provide evidence that AdipoR-1 levels are inversely correlated with the biologically malignant phenotypes of endometrial carcinoma, and that endometrial cancer patients with positive expression of AdipoR-1 experience an improved prognosis in terms of progression-free and overall survival.

In conclusion, the present study demonstrated the expression of AdipoR-1, AdipoR-2 and ObR in human endometrioid adenocarcinoma of the uterine corpus. In addition, the decreased expression of AdipoR-1 molecules was associated with tumour growth, invasion and metastasis, representing factors predictive of poor prognosis in patients with endometrial carcinoma. Furthermore, adiponectin-based anticancer therapies may prove useful for treating endometrial cancer.

## Figures and Tables

**Table I:** Characteristics of 77 patients with endometrioid adenocarcinoma of the endometrium.

Characteristics	n
Stage	
IA	30
IB	23
II	9
IIIA	2
IIIC	13
Grade	
G1	39
G2	22
G3	16
Myometrial invasion	
<1/2	38
≥1/2	39
Cervical stromal invasion	
Negative	59
Positive	18
Adnexal invasion	
Negative	69
Positive	8
Lymphovascular space involvement	
Negative	52
Positive	25
Lymph node metastasis	
Negative	65
Positive	12
Peritoneal cytology	
Negative	61
Positive	16
Body mass index, kg/m^2^	
<25	45
≥25 to <30	16
≥30 to <35	9
≥35	7
Adjuvant therapy	
None	26
Radiotherapy	12
Chemotherapy	39
AP	9
TJ	30
Age, years (mean ± SD)	56.91±9.98

AP, doxorubicin + cisplatin; TJ, paclitaxel + carboplatin.

**Table II:** Clinicopathological characteristics in relation to the immunohistochemical expression of AdipoR-1, AdipoR-2 and ObR in tumours obtained from 77 patients with endometrial carcinoma.

Clinicopathological characteristics	n	AdipoR-1, n (%)	AdipoR-2, n (%)	ObR, n (%)
Stage				
IA	30	23 (76.7)	18 (60.0)	10 (33.3)
IB	23	14 (60.9)	14 (60.9)	13 (56.5)
II	9	6 (66.7)	6 (66.7)	4 (44.4)
IIIA	2	0 (0.0)	2 (100)	0 (0.0)
IIIC	13	3 (23.1)	7 (53.8)	6 (46.2)
		P=0.0073	P=0.7920	P=0.3507
Grade				
G1	39	34 (87.2)	27 (69.2)	18 (46.2)
G2	22	10 (45.5)	14 (63.6)	13 (59.1)
G3	16	2 (12.5)	6 (37.5)	2 (12.5)
		P<0.0001	P=0.0867	P=0.0138
Myometrial invasion				
<1/2	38	29 (76.3)	24 (63.2)	14 (36.8)
≥1/2	39	17 (43.6)	23 (59.0)	19 (48.7)
		P=0.0051	P=0.8162	P=0.3594
Cervical stromal invasion				
Negative	59	38 (64.4)	35 (59.3)	25 (42.4)
Positive	18	8 (44.4)	12 (66.7)	8 (44.4)
		P=0.1720	P=0.7831	P=0.8765
Adnexal invasion				
Negative	69	46 (66.7)	42 (60.9)	31 (44.9)
Positive	8	0 (0.0)	5 (62.5)	2 (25.0)
		P=0.0004	P=0.9287	P=0.4544
Lymphovascular space involvement				
Negative	52	39 (75.0)	35 (67.3)	25 (48.1)
Positive	25	7 (28.0)	12 (48.0)	8 (32.0)
		P=0.0001	P=0.1360	P=0.2235
Lymph node metastasis				
Negative	65	44 (67.7)	41 (63.1)	27 (41.5)
Positive	12	2 (29.2)	6 (50.0)	6 (50.0)
		P=0.0024	P=0.5216	P=0.7526
Peritoneal cytology				
Negative	61	37 (60.7)	36 (59.0)	27 (44.3)
Positive	16	9 (56.3)	11 (68.8)	6 (37.5)
		P=0.7805	P=0.5722	P=0.7785
BMI, kg/m^2^				
<25	45	27 (60)	29 (64.4)	20 (44.4)
≥25	32	19 (59.4)	18 (56.3)	13 (40.6)
		P=0.9560	P=0.4872	P=0.8172
Total	77	46 (59.7)	47 (61.0)	33 (42.9)

AdipoR-1, adiponectin receptor type 1; AdipoR-2, adiponectin receptor type 2; ObR, leptin receptor; BMI, body mass index.

**Table III:** Uni- and multivariate analyses of variables associated with progression-free survival using the Kaplan-Meier method with log-rank test, logistic regression model and Cox proportional hazard model.

		Kaplan-Meier method		Univariate analysis			Multivariate analysis		
				
Variables	n	Five-year survival rate, %	P-value	HR	95% CI	P-value	HR	95% CI	P-value
Stage									
I	53	92.10	<0.0001	−1.012	0.218–0.608	0.0001	−0.554	0.130–2.540	0.4648
II/III	24	49.20							
Grade									
G1/G2	61	88.50	<0.0001	−1.934	0.055–0.381	<0.0001	−0.124	0.397–1.967	0.7615
G3	16	43.80							
Myometrial invasion									
<1/2	38	92.10	0.004	−1.642	0.056–0.675	0.0099	0.302	0.675–2.708	0.394
≤1/2	39	66.20							
Cervical stromal invasion									
Negative	59	87.80	0.00001	−1.724	0.066–0.483	0.0007	−0.457	0.222–1.808	0.3932
Positive	18	48.60							
Adnexal invasion									
Negative	69	86.50	<0.0001	−2.957	0.017–0.154	<0.0001	−2.086	0.030–0.508	0.0037
Positive	8	12.50							
Lymphovascular space involvement									
Negative	52	85.90	0.0216	−1.067	0.133–0.893	0.0283	−0.005	0.495–1.998	0.9881
Positive	25	63.80							
Lymph node metastasis									
Negative	65	90.40	<0.0001	−2.921	0.018–0.157	<0.0001	−1.953	0.040–0.509	0.0027
Positive	12	12.50							
Peritoneal cytology									
Negative	61	81.60	0.237	−0.623	0.188–1.531	0.2445	0.635	0.605–5.886	0.2741
Positive	16	67.50							
AdipoR-1 expression									
Negative	31	59.90	0.0004	1.780	1.928–18.246	0.0019	−0.068	0.454–1.921	0.8525
Positive	46	91.20							
AdipoR-2 expression									
Negative	30	75.90	0.9722	0.017	0.384–2.697	0.9722	−0.363	0.361–1.340	0.2779
Positive	47	80.90							
ObR expression									
Negative	44	79.20	0.945	0.034	0.393–2.723	0.945	−0.218	0.409–1.580	0.5273
Positive	33	78.30							
BMI, kg/m^2^									
<25	45	82.20	0.3527	0.126	0.716–1.797	0.5907	−0.069	0.570–1.529	0.7834
≥25	32	74.00							
Age, years									
<60	45	79.50	0.9015	−0.042	0.606–1.516	0.8567	0.083	0.549–2.151	0.8121

HR, hazard ratio; CI, confidence interval; AdipoR-1, adiponectin receptor type 1; AdipoR-2, adiponectin receptor type 2; ObR, leptin receptor; BMI, body mass index.

**Table IV:** Uni- and multivariate analyses of variables associated with overall survival using the Kaplan-Meier method with log-rank test, logistic regression model and Cox proportional hazard model.

		Kaplan-Meier method		Univariate analysis			Multivariate analysis		
				
Variables	n	Five-year survival rate, %	P-value	HR	95% CI	P-value	HR	95% CI	P-value
Stage									
I	53	100	<0.0001	−0.894	0.244–0.687	0.0007	−0.871	0.097–1.808	0.2431
II/III	24	70.80							
Grade									
G1/G2	61	95.1	0.0101	−1.575	0.055–0.777	0.0196	0.655	0.838–4.422	0.1228
G3	16	75.0							
Myometrial invasion									
<1/2	38	100	0.0177	−2.106	0.015–0.974	0.0472	0.208	0.616–2.461	0.5566
≥1/2	39	82.10							
Cervical stromal invasion									
Negative	59	94.90	0.0078	−1.653	0.049–0.741	0.0166	0.837	0.748–7.123	0.1454
Positive	18	77.80							
Adnexal invasion									
Negative	69	98.60	<0.0001	−3.867	0.004–0.108	<0.0001	−2.266	0.032–0.340	0.0002
Positive	8	25.0							
Lymphovascular space involvement									
Negative	52	96.20	0.0607	−1.156	0.088–1.125	0.0752	0.192	0.577–2.545	0.6125
Positive	25	80.0							
Lymph node metastasis									
Negative	65	100	<0.0001	−4.510	0.001–0.093	<0.0001	−1.193	0.107–0.860	0.0248
Positive	12	41.70							
Peritoneal cytology									
Negative	61	93.40	0.0368	−1.313	0.072–1.009	0.0515	−0.109	0.332–2.425	0.8306
Positive	16	81.30							
AdipoR-1 expression									
Negative	31	77.40	0.0018	2.565	1.620–10.427	0.0158	0.026	0.478–2.202	0.947
Positive	46	100							
AdipoR-2 expression									
Negative	30	86.70	0.3374	0.637	0.504–7.092	0.3453	0.387	0.796–2.726	0.2176
Positive	47	93.60							
ObR expression									
Negative	44	90.90	0.3605	−0.608	0.145–2.043	0.3676	−0.432	0.321–1.312	0.2284
Positive	33	90.90							
BMI, kg/m^2^									
<25	45	91.10	0.2739	0.095	0.696–1.737	0.685	0.034	0.610–1.754	0.8988
≥25	32	90.60							
Age, years									
<60	45	91.10	0.9348	−0.024	0.617–1.546	0.9186	−0.192	0.417–1.636	0.5828
≥60	32	90.60							

HR, hazard ratio; CI, confidence interval; AdipoR-1, adiponectin receptor type 1; AdipoR-2, adiponectin receptor type 2; ObR, leptin receptor; BMI, body mass index.
